# Supplementary material for: Are carbon emissions trading and green financial instruments synergistic? -Comprehensive quantitative research based on content analysis
Source: PLoS One. 2024 Mar 7;19(3):e0298601. doi: 10.1371/journal.pone.0298601 (PMC10919601; doi:10.1371/journal.pone.0298601)
Supplement: S1 Data — (ZIP) [file pone.0298601.s001.zip › Data/地级市Z_F工作报告.zip!/╕╩╦α/╞╜┴╣╩╨/└┤╫╘╣1⁄2╓┌║┼í╛┬φ┐╦╩2╛▌═°í┐_2019.docx]

各位代表：

现在，我代表市人民政府向大会报告工作，请予审议，并请各位政协委员和列席人员提出意见。

2018年工作回顾

2018年，面对稳中有变的经济形势、频发重发的自然灾害和繁重艰巨的工作任务，在市委的坚强领导下，在市人大、市政协的监督支持下，我们认真贯彻习近平新时代中国特色社会主义思想和党的十九大精神，全面落实习近平总书记视察甘肃重要讲话和“八个着力”重要指示精神，按照“党建统领，‘四化’统筹，交通先行，产业支撑，决战脱贫，决胜小康，建设绿色开放幸福美好新平凉”的发展思路，对标高质量发展要求，积极应对困难挑战，聚力冲刺脱贫攻坚，统筹推进重点工作，全市经济稳中有进，社会大局和谐稳定，较好地完成了市四届人大二次会议确定的目标任务。全市实现生产总值395.17亿元，增长2.1%；规模以上工业增加值52.25亿元，增长2.6%；社会消费品零售总额208.11亿元，增长6.6%；一般公共预算收入30.47亿元，同口径增长11.7%，财政支出完成212.7亿元，增长15.7%；城乡居民人均可支配收入达到27448元和8303元，分别增长8%和9.1%。

（一）实施“九大冲刺行动”，脱贫攻坚取得决定性进展。以产业扶贫为核心，坚持“远抓苹果近抓牛、当年脱贫抓劳务”， “一户一策”推动政策措施到村到户到人，全市脱贫8.65万人，退出贫困村230个，崆峒区脱贫摘帽，华亭、泾川、灵台、崇信4县（市）将达到脱贫摘帽标准，贫困发生率下降到4.63%，创造了脱贫攻坚的“平凉模式”，庄浪县获得全国脱贫攻坚组织创新奖。推动龙头带动全覆盖，市级财政列支产业发展专项资金3000万元，争取国省专项扶贫资金9.3亿元，培育市级以上龙头企业200户，70%的贫困群众嵌入产业链。推动“三变”改革全铺开，探索推广了“庄浪模式” “静宁模式”等改革模式，全市76%的行政村开展了“三变”改革，新登记注册农民专业合作社1332个，3.67万农户入股分红3450万元，150个村集体获益953万元，进一步拓宽了农民增收渠道，消除了“空壳村”。推动金融保险全加入，累计发放各类扶贫贷款58亿元，持续扩大农业政策性保险覆盖面，有效解决了贫困群众发展资金难题，防范了自然、市场双重风险。推动危旧住房全清零，易地扶贫搬迁3813户，改造农村危房8709户，D级危房全部清零，C级危房基本消除，提前一年完成农村存量危房改造任务，走在全省前列。推动劳动就业全对接，总结推广“灵台模式”，兴办扶贫车间，有组织输转贫困劳动力3.6万人，公益性岗位安置贫困群众8906人，就业扶贫经验在全省推广。推动教育医疗全跟进，陕甘宁三省区医养中心和职业教育高地建设扎实推进，落实各类教育扶贫资金4.1亿元，资助学生30万人（次），建档立卡贫困人口合规医疗费用报销比例达到85%。全国唯一的教育精准扶贫国家级示范区在平凉先行先试，省上召开了现场会，教育部向全国推广了主要做法。推动社会救助全兜底，为24.3万城乡低保对象发放保障金6亿元，为8416名城乡特困供养对象发放供养资金5111万元，实现了应扶尽扶、应保尽保。推动党政干部和社会各界全参战，压紧压实各级责任，引导社会组织、爱心企业和人士积极参与，加强东西部协作，落实帮扶资金9亿元，形成了全社会参与脱贫攻坚的强大合力。推动党建统领全保障，深入开展“一强三创”行动，充分发挥“三个带头人”作用，全面推行“四链”模式，基层组织引领和保障脱贫攻坚的战斗堡垒作用得到有效发挥。

（二）突出交通先行，多措并举争建项目。大力推行“三个一”包抓责任制和“三个清单”管理制度，全市实施500万元以上项目489个，完成投资215亿元，固定资产投资降幅比上年收窄18.86个百分点。着眼长远发展抓谋划，市县两级财政列支7654万元、争取国省900万元的项目前期经费，运用“八个一批” “四个渠道”方式方法筛选论证项目，多次赴省进京衔接汇报，备受关注的平凉机场、平庆高铁、天平动车、陇东电力外送通道等项目前期取得积极进展，市体育运动公园、平凉一中新校区、天门塬新区综合开发等项目前期加快推进。立足稳定增长抓建设，争取中央和省级预算内投资项目203项11.3亿元，扎实开展项目审批“百日攻坚行动”和集中开工、观摩督查等活动，平天、彭大、静庄、华亭至泾源4条高速加快实施，平华一级等7条普通省道积极推进，开建县乡道改造工程210公里，引洮供水二期静宁骨干工程、配套工程和新集水库加快建设，平凉750千伏变电站配套送出工程建成运行。围绕扩张总量抓招商，坚持上下联动，先后赴京沪、粤港等地精准招商，举办平凉红牛产业香港专场招商推介会，组团参加国际进口博览会、敦煌文博会、兰洽会等节会，与中纺机、陕旅等行业领军企业达成了一批合作协议，成功引进福建云计算区块链数据中心、台湾桦晨电子等项目，庄浪印象水洛城等项目加快建设。全市新开工招商项目305项，到位资金增长6.1%。

（三）坚持“四化”统筹，下大力气推动新旧动能转换。以推进供给侧结构性改革为主线，以“三重”“三一”为抓手，打造新的产业集群，加快“四化”进程。扎实推进农业现代化，举办平凉红牛发展战略研讨会，中国农科院西部肉牛种质科技创新基地落户平凉，葫芦河流域国家级果品高新技术示范区、泾汭河川区国家级蔬菜产业园建设加快推进，培育了庙川、鼎康规模养殖场，大潘、东庄、齐翔现代农业示范园，德美冷链物流园、华亭中药材交易市场等一批产加销龙头企业，新增家庭农场166个，流转土地108万亩，牛饲养量净增10万头，苹果在受灾之年减产不减收，蔬菜产量达到46.6万吨，“三品一标”农产品占到54%，“平凉红牛”“平凉金果” 拿到了供应国家级运动员及重大赛事的“入场券”，海升超越农业串收番茄在德国柏林荣获世界番茄最具创新奖，我市被命名为全国首批两个苹果优质苗木繁育基地之一。全市第一产业增加值完成87.89亿元。着力推进新型工业化，坚持煤电产业与非煤产业两手抓，煤电化冶产业发展规划通过省上评审，赤城煤矿、20万吨聚丙烯加快推进，邵寨煤矿通过核准评审，五举煤矿产能置换方案获批，新河400万吨、兴盛300万吨建筑石料、庆华陶瓷新型建材、宏达淀粉水蛋白提取等项目建成投产，天纤棉业20万锭棉纱、永成中药饮片等项目加快实施，全市工业用电量保持两位数增长。平凉海螺水泥荣获2018年中国最具成长性建材企业100强，平凉启航未来智能终端光电产业园等高新技术产业项目落地，工业转型升级迈出了新步伐。加快推进新型城镇化，编制完成《城市发展战略规划》《泾河水系景观规划》等12项规划，出台中心城区资源资产经营管理意见，闲置建设用地处置、基础设施和公用事业特许经营等工作加快推进，棚户区改造扭转被动局面。投资63亿元，实施中心城区建设项目10大类50项，热电联产集中供热二期建成运营、新增供热面积205万平方米，崆峒大道改造、泾河北路西延伸段及宏达国盛、景海商贸城等工程建成，六县（市）城区和重点小城镇基础设施、公共服务项目加快推进，华亭撤县设市，全市乡改镇10个，城市综合承载能力和聚集效应不断增强，新增城镇人口2.4万人，城镇化率41.13%。大力推进信息化，智慧城市运营指挥中心、地理信息系统、“雪亮工程”进展顺利，城乡网络实现增宽提速，村村通光纤改造被工信部树立为全国典型。5个国家级电子商务进农村综合示范县加快建设，乡级电商服务站、村级服务点扩面提质。全市第三产业增加值203.39亿元，增长4.8%。

（四）强化底线红线意识，千方百计改善生态环境。扎实开展大气、水、土壤污染防治行动，认真整改环保督察反馈问题，投资2.97亿元，完成平凉电厂、崇信电厂3台机组超低排放改造，清理整治燃煤锅炉499台，建成覆盖城乡的优质煤配送网络，居民清洁取暖改造3万户，中心城区环境空气优良天数比率为91.6%；全面推行河（湖）长制，积极应对环境突发事件，投资1.05亿元，3个工业园区工业污水处理厂和中心城区、泾川县城区污水处理厂提标扩容工程投运，建成重点乡镇污水处理设施29个，泾河、汭河、达溪河及15个县级以上集中式饮用水水源地水质稳定达标；深入开展全域无垃圾治理，19个农村环境综合整治、8个畜禽养殖污染防治项目全部完成，中心城区、华亭、灵台、静宁城区生活垃圾填埋场二期和34个重点乡镇垃圾处理设施加快建设，抓建美丽乡村83个、万村整洁村220个。积极推动绿色发展崛起，“1+1+10+X”生态产业框架体系基本建立，实施5000万元以上十大生态产业项目77项，崆峒区列为全国50个资源循环利用基地之一。大力实施城乡增绿扩绿工程，造林绿化53.5万亩，陇东百万亩土地整治工程全面完成，全省大规模国土绿化暨退耕还林建设、生态文明建设示范区创建现场会在我市召开。

（五）紧盯重点领域关键环节，持续推进改革开放。深化“放管服”改革，市县两级政务服务中心建设取得突破性进展，90%以上政务服务事项进驻中心，850项实现“最多跑一次” ，企业开办时间控制在5个工作日以内，“市区一体、同址并窗、一号受理、一窗办理”的政务服务模式得到省上肯定。“多证合一”“一照一码”登记制度全面推行，“双随机一公开”监管范围扩大，降低企业用电成本5097万元，为各类市场主体减税让利8.6亿元，政策效应加速显现，新增非公经济市场主体1.78万户。平凉工业园区体制机制改革全面到位，开展排污权交易试点，配售电有限公司挂牌成立，获批省级高新技术产业开发区。华亭工业园区循环化改造示范试点通过国家验收，泾川工业集中区升级为省级开发区。出台基本公共服务领域财政事权和支出责任划分改革方案，制定防范化解政府债务和隐性债务风险实施意见，政府依法理财和依规举债行为进一步规范。引进黄河财产保险等金融机构5家，省金控集团平凉融资担保公司注册运营，建成全省首批苹果期货交割仓库，在全省率先实现县（市、区）农商银行全覆盖，全市贷款余额增长5.82%，社会融资达到70亿元。平凉海关获批设立，国家级出口食品农产品质量安全示范区创建通过省级验收，全市进出口总额4.5亿元，增长120%。文化旅游市场化改革迈出坚实步伐，精心筹备、高端策划，参加央视《魅力中国城》竞演，荣获优秀魅力城市、年度魅力文旅扶贫城市称号，崆峒山被评为年度魅力文化景区，在国家大平台展示了平凉形象，打响了“问道崆峒·养生平凉”品牌。调整崆峒山大景区管理体制，引进战略投资者合作经营开发崆峒山、大云寺·王母宫大景区，吸引社会资本开发乡村旅游，实施旅游重点项目36个，新增3A级景区4个；成功举办央视书画频道走进平凉、道教名山围棋邀请赛、崆峒—武当武术交流展示、万人太极拳展演等崆峒文化旅游节系列活动，平凉荣获中国国家旅游年度榜单最佳全域旅游目的地称号，全市旅游接待人数、综合收入分别增长9.6%和4.8%。

（六）加快补齐短板，用心用力保障和改善民生。财政用于民生的支出占总支出的83.8%。改造老旧住宅639幢，惠及2.4万户8万余人，投放新能源公交车105辆，人居环境极大改善，城市“颜值”不断提升。认真落实“双创”政策，新增城镇就业3.35万人，输转城乡劳动力48万人，创劳务收入99亿元。全面落实各项提标政策，35.9万城乡居民和企业职工享受养老金17.3亿元，为60户企业5.1万名职工发放稳岗补贴1894.7万元。大力发展教育事业，新建改扩建幼儿园46所，“改薄”项目进展顺利，全市整体实现义务教育基本均衡发展目标，崇信县小班化教学模式在全国介绍了经验，高中教育质量稳步提升。坚持“三医”联动，分级诊疗制度逐步完善，市县级公立医院全部实现药品零差率销售，重特大疾病医疗救助病种由26种扩大到50种，为40.2万人（次）报销住院医疗费10.8亿元。加快公共文化服务体系建设，市博物馆新馆陈列布展，一批乡村（社区）综合性文化服务中心和体育健身工程建成。扎实落实防灾减灾措施，市县两级投入1600万元，有效应对洪涝、冰雹、低温冻害等自然灾害，最大限度减少了灾害损失。严格落实安全生产责任制，切实强化食品药品安全监管。扎实开展扫黑除恶专项斗争，全面推进全国禁毒示范城市创建，严厉打击各类违法犯罪活动，社会大局持续稳定。我市再次荣膺甘肃省文明城市称号。

同时，民族宗教、统计调查、公共资源管理、档案、外事侨务、地震、气象、人防、地方志、红十字、老龄、残疾人、关爱留守儿童等事业取得新的成效，双拥共建、国防动员、民兵预备役等工作深入开展。

过去的一年，我们切实提高政治站位，着力加强政府自身建设，全面落实党中央、国务院和省委、省政府及市委重大决策部署，坚决整改中央和省委巡视反馈问题。自觉接受人大法律监督、工作监督和政协民主监督，提请市人大常委会审议了《平凉市物业管理条例》等地方性法规，78件人大代表建议、112件政协委员提案全部办结。积极推进政府职能转变，加强意识形态阵地建设和管理，严格执行政府工作规则和重大事项决策程序，积极应对行政诉讼，法治政府建设进程加快。坚决落实中央八项规定及实施细则精神，深入开展“转变作风改善发展环境建设年”活动，从严执行省政府“十不准”规定，各级干部作风不断改进、政府效能明显提升，涌现出一批全国、全省先进典型。严格履行全面从严治党主体责任，政府系统党风廉政建设和反腐败工作扎实推进。

各位代表，一年来，全市经济社会发展取得的成绩来之不易，这是省委、省政府和市委坚强领导的结果，是市人大、市政协监督支持的结果，更是全市人民团结奋斗的结果。在此，我代表市人民政府，向辛勤工作在各条战线的广大干部群众，向中央、省驻平单位和驻平部队，向各民主党派、工商联、无党派人士，向广大民营企业家，向离退休老同志，向关心、支持平凉改革发展和脱贫攻坚的各界人士，致以崇高的敬意和衷心的感谢！

总结成绩的同时，我们也清醒地认识到，全市经济社会发展还面临不少困难和问题，政府工作与人民期盼仍然存在不小差距。一是受内外部环境影响，经济指标波动性大、稳定性弱，地区生产总值、规上工业增加值等指标没有达到预期，固定资产投资仍未实现由负转正，巩固经济回稳向好势头任务艰巨。二是经济结构不优，主导产业总量不大、特色产业层次不高，仅有的67户规上工业企业退出16户，新上煤电项目核准受限，现有企业不能满负荷生产，工业经济普遍运行困难；农业“初字号”“原字号”产品占比大，龙头企业少小弱，产业化程度低；文化旅游资源整合不够，产业融合度不高，市场化运营滞后。全市非公经济增加值占地区生产总值的48.8%，民间投资占全市投资总量的39.1%，均低于全国、全省平均水平。三是深度贫困片带脱贫难度大，增收产业尚处于培育成长阶段，贫困户家庭经营收入还不够稳定，村级集体经济比较薄弱，扶志扶智还有待进一步加强。四是道路交通、生态环境等方面基础设施短板明显，成为全市人民高度关注并迫切希望解决的热点痛点问题。五是城镇功能布局不够合理，基础设施配套不完善，出行难、停车难、如厕难等“城市病”突出，特色小镇建设滞后，城镇产业培育不足，吸纳就业能力弱，市场化经营城市的机制还未形成，城镇化率低于全省6.56个百分点。六是个别政府部门和干部思想保守、精神不振、担当不够、效率低下，抓项目促发展的劲头不足，“放管服”改革还不到位，政务服务互联互通水平比较低，政策落实效果不够明显，营商环境亟待改善。对此，我们将采取更加有力的措施，认真研究解决。

2019年工作总体要求和主要任务

2019年是新中国成立70周年，是决胜全面建成小康社会关键之年。面对经济下行压力加大、外部环境发生深刻变化的复杂形势，我们要牢牢抓住国家新一轮西部大开发带来的战略机遇、“一带一路”建设带来的开放机遇、乡村振兴战略实施带来的政策机遇、东部产业加速向西部转移带来的市场机遇、加快绿色发展带来的生态机遇，坚持目标导向、问题导向、创新导向和实干导向，突出党建统领“统”的要求，保持推动发展“稳”的定力，增强追赶跨越“进”的信心，强化改革创新“闯”的劲头，展现履责担当“新”的作为，脚踏实地、主动出击，全力推动经济高质量发展。

政府工作总的要求是：以习近平新时代中国特色社会主义思想为指导，全面贯彻党的十九大和十九届二中、三中全会精神，统筹推进“五位一体”总体布局，协调推进“四个全面”战略布局，深入落实习近平总书记视察甘肃重要讲话和“八个着力”重要指示精神，坚持稳中求进工作总基调，坚持新发展理念，坚持推动高质量发展，坚持以供给侧结构性改革为主线，坚持深化市场化改革、扩大高水平开放，按照市委四届四次全会确定的总体思路，统筹推进稳增长、促改革、调结构、惠民生、防风险工作，全面打赢脱贫攻坚战，保持经济持续健康发展和社会大局稳定，为全面建成小康社会收官打下决定性基础，以优异成绩庆祝中华人民共和国成立70周年。

经济社会发展主要预期目标是：地区生产总值增长6%以上，规上工业增加值增长6.5%，固定资产投资增长8%，社会消费品零售总额增长8%，一般公共预算收入同口径增长6%，城乡居民人均可支配收入分别增长7%和8%，城镇新增就业3万人，居民消费价格指数控制在103%左右，单位生产总值能耗和主要污染物排放完成省上下达的控制目标。

重点做好八个方面的工作：

（一）聚焦深度贫困乡村，举全市之力决战决胜脱贫攻坚。把脱贫攻坚作为全面建成小康社会最大的硬任务，作为实施乡村振兴战略第一位的任务，深入开展打赢脱贫攻坚战三年行动，持续推进“九大冲刺行动”，精准施策，攻坚拔寨，确保庄浪、静宁两县脱贫摘帽，7.16万贫困人口脱贫退出，全市实现整体脱贫。

充分发挥产业扶贫带动作用。坚持“远抓苹果近抓牛、当年脱贫抓劳务”，扎实落实“一户一策”，深入推进农业供给侧结构性改革，大力发展现代丝路寒旱农业，加快构建现代农业产业体系，严格落实粮食安全省长责任制，积极推进“粮改饲”，全年粮食产量稳定在100万吨以上；深入实施“牛扩量、果转型、菜提质”工程，突出肉牛规模化养殖、果园更新换代、蔬菜周年均衡供应，新建养殖小区30个，牛饲养量净增10万头以上，新植矮化密植园4.2万亩，静宁、庄浪进入全省40万亩苹果产业大县行列，蔬菜种植面积70万亩。加快构建现代农业生产体系，推进中国农科院西部肉牛种质科技创新基地建设，每个县（市、区）建立500头规模的育种群，肉牛品种改良15万头，创建果品标准化生产等认证基地35万亩，加快建设“两带六片”蔬菜产业集中区，新增设施蔬菜6000亩。加快构建现代农业经营体系，坚持自建与引进并举，新建改扩建龙头企业25户；放大“庄浪模式”“静宁模式”带动效应，实施农民专业合作社能力提升工程，清理注销“空壳社”，壮大村级集体经济，培育家庭农场100个、农民合作社联合社30个，促进小农户与现代农业发展的有机衔接。推进政策性农业保险增品扩面、提标降费，全面开办种养产业综合保险，行政村金融综合服务室全覆盖。大力推广“灵台模式”，积极对接市场需求，精准开展技能培训，全市新增“扶贫车间” 77个，输转劳务47万人，创收105亿元。统筹推进电商、旅游、光伏扶贫，扶持发展小家禽、小庭院、小作坊、小手工、小买卖“五小”产业。全市第一产业增加值完成95亿元。

加快补齐“两不愁三保障”短板弱项。实施农村饮水安全巩固提升工程10处，解决10万人饮水不稳定问题。加强农村危房动态管理，兜底解决贫困户新出现危房。全面落实教育扶贫、健康扶贫各项政策，加强义务教育控辍保学工作，大力推进教育精准扶贫国家级示范区平凉先行先试，巩固提升医疗报销比例和保障救助水平，确保每一个学生不因贫困上不起学、每一个贫困群众不再为看病发愁。统筹推进贫困村基础设施建设，完成县乡道改造90公里、安全生命防护工程1000公里、“畅返不畅”路段整治100公里，改造升级乡镇、村综合文化服务中心。

持续完善脱贫攻坚保障机制。严格落实脱贫攻坚责任制，加强贫困人口动态管理，探索解决收入略高于建档立卡贫困户的群体缺乏政策支持问题。加大资金投入，新增财力、结余结转资金、整合涉农资金优先用于脱贫攻坚。强化扶贫资金常态化监管，持续开展扶贫领域腐败和作风问题专项治理。坚持扶贫与扶志、扶智相结合，教育引导贫困群众树立脱贫光荣意识，自力更生，脱贫致富。深化定点扶贫、东西扶贫协作帮扶机制，鼓励各类社会组织开展到村到户精准扶贫，构建大扶贫格局。

统筹实施乡村振兴战略。坚持农业农村优先发展，一体谋划乡村振兴和脱贫攻坚，加快编制乡村振兴总体规划和专项规划。推进新一轮农村改革，完善农村土地确权后续工作，全面推开农村集体产权制度改革。加强耕地和永久基本农田保护，彻底清理整治“大棚房”，遏制农地非农化。以整市创建全省现代农业产业园为载体，加快建设葫芦河流域国家级果品高新技术示范区、泾汭河川区国家级蔬菜产业园、静宁县国家级现代农业产业园，改造提升农业生产能力，改革优化种养模式，健全农产品加工、冷链、仓储、物流体系，加快两个“三品一标”建设，推进“平凉红牛”全国驰名商标创建和国家地理标志认证，打造全产业链全价值链特色产业集群。全力改善农村人居环境，开展农村厕所、农村垃圾、农村风貌“三大革命”，推进农村生活污水治理、废旧农膜回收利用与尾菜处理、农村畜禽养殖废弃物及秸秆资源化利用“三大行动”，完成农村户用厕所改造5万户以上，70%的乡镇街道、建制村至少建成1处无害化公厕，有效治理乱搭乱建、乱堆乱放、畜禽乱窜等问题，建成美丽乡村示范村60个。健全村民共建共治体系，推行村级事务阳光工程，深化治理高价彩礼推动移风易俗专项行动，拓展群众性精神文明创建活动，大力培育文明乡风、良好家风、淳朴民风。

（二）发挥项目带动作用，全力以赴扩投资稳增长。坚持以投资结构引导经济结构，争建并举、大小齐抓、质效兼顾，全市实施500万元以上项目396项，固定资产投资达到232亿元以上。

高标准谋划项目。坚持 “八个一批”谋划争取项目和 “四个渠道”生成运作项目的工作方法，积极对接国省基础设施补短板、投融资改革、增加地方政府专项债券规模等政策措施，完善项目前期费用逐年增长机制，围绕高质量发展、绿色发展，谋划一批生态产业上中下游全链条项目；着眼解决经济社会发展中最为紧迫、最为急需的突出问题，谋划一批道路交通、水电通讯、防灾减灾等重大基础设施项目和人工智能、工业互联网、物联网等新型基础设施建设项目；紧盯社会需求，谋划一批幼教职教、医疗卫生、文化旅游、健康养老等民生项目。做深做细项目前期，争取更多项目进入国省盘子，项目储备库动态保持在3000亿元以上。

大力度推进项目。突出交通先行，加快平庆高铁前期，力争平凉机场年内具备开工条件，灵华高速3月份全线进场，推进平天、彭大、静庄、华亭至泾源高速和国道312线改造；落实市县事权责任，持续推进平华一级、彬县至华亭、平峰至八里等7条省道建设。加快白龙江引水、崆峒水库改扩建、泾川盘口水库等项目前期，推进引洮供水二期静宁受水区配套工程、灵台新集水库等在建项目。实施脱贫攻坚农网改造工程，静宁成纪330千伏送变电工程竣工投运，110千伏配套送出工程建成。坚持一个项目一套班子，平凉一中新校区、平凉万达广场一季度开工，市体育运动公园上半年开工。

多渠道招引项目。成立市政府招商团队，完善招商引资政策措施，采取以商招商、点对点招商、委托代理招商等多种方式，引项目与引资本并举，引进企业与企地合作兼顾，签约实施一批龙头型、基地型、产业型、财源型项目，切实提高招商引资的质量效益。积极对接洽谈，强化跟踪服务，抓好江苏乐能10万吨锂电池、上海森鑫碳纳米管、上海千牧大数据中心等项目落地建设。全市实施招商引资项目300项以上，到位资金增长8%。

全方位保障项目。加强要素供给，增强承接能力，打通项目落地“最后一公里”。推行国有资产出让和资产证券化等融资新模式，积极争取政策性、开发性、商业性金融机构支持，发挥惠民、泓源、富民、文旅投等投融资平台作用，促使重点项目扩大股权和债券融资，稳步推行交通领域“四种投融资模式”，充分利用政府债券、PPP、产业基金模式，保障项目建设资金需求。用足用活国家和省上支持深度贫困地区用地倾斜政策，推进城乡建设用地增减挂钩节余指标交易，加大闲置低效用地盘活力度，提高土地投资强度。坚持项目督查观摩常态化，形成抓建项目合力动力压力，促使项目快落地快建设快见效。

（三）构建生态产业体系，打造高质量发展新引擎。持续深化供给侧结构性改革，贯彻落实“巩固、增强、提升、畅通”方针，着力优化存量、扩大增量、提升质量，推动绿色发展崛起，培育新的经济增长极，加速新旧动能转换。

做大做强绿色煤电产业。围绕建设平凉煤电化冶“双千亿”产业基地，按照“稳步扩煤、加快转电、突破化工”的思路，全力加快煤电化冶重大项目建设，推动陇东电力外送通道工程核准，复工建设邵寨煤矿、五举煤矿，建成赤城煤矿、黄庄煤矿，加快推进唐家河煤矿前期。实施华煤等企业绿色化改造，发展高精度煤炭洗选加工，鼓励煤矿和选煤厂开展系统节能，推行清洁生产。支持企业延链补链扩链，20万吨聚丙烯项目试车生产，40万吨煤制乙二醇项目前期取得突破性进展。

转型提升传统产业。巩固“三去一降一补”成果，建设工业大数据平台，实施规上企业分类管理，加快淘汰落后产能，“一企一策”帮扶停产减产企业恢复生产，有序退出“僵尸企业”。加快实施中国制造2025平凉发展行动，推动互联网、大数据、人工智能和实体经济深度融合，开展装备制造和新型建材产业智能化、绿色化、循环化改造行动，大力发展新型装配式建筑材料，推广应用绿色环保建材，力争兴泰100万吨建筑石料项目建成投产，支持庆华陶瓷提质增效，建设安口陶瓷产业园。支持大唐丰泰与中纺机、中棉集团建立战略合作关系，加快规划设计，优化组合资源资产，采取设备租赁等模式，“保姆式”推动50万锭棉纱生产、棉花储备库及物流园建设，加快打造西北纺织城。实施农产品加工业提升行动，吸引社会力量发展果汁、淀粉、肉制品、中药材加工等“农字号”企业，推进陇香3万吨食醋、2万吨食用油和惠丰10万吨石磨面粉生产线建设，建成永成中药饮片生产基地、银旺中药复合颗粒生产线等项目，农产品加工转化率达到54%。

培育壮大战略性新兴产业。采取轻资产、园中园模式，支持启航未来投资集团（香港）有限公司投资25亿元，加快建设平凉启航未来智能终端光电产业园，当年入驻企业15家以上，培育和发展以生产液晶显示屏、触摸屏、摄像头、无人机等为主的智能光电产业生态链基地，通过3年努力，带动就业5000人以上，实现产值100亿元以上。强化新能源应用，大力推广甲醇汽车、电动汽车，推进充电站、充电桩配套建设。

融合发展文旅产业。围绕创建国家全域旅游示范市，推进景区资源“三权分置”、提质扩容，引资引智壮大旅游市场主体，加快陕旅、省城投与崆峒山、大云寺·王母宫大景区合作开发运营，实施静宁35度苹果景区、海寨沟、文化产业大厦等重点项目48个，推动大云寺·王母宫大景区创建5A级景区，建成4A级景区1处、3A级景区2处，提升泾川凤凰等16个省级乡村旅游示范村建设层次。加强景区景点道路连接线、游客集散中心、旅游厕所等基础设施建设，提升配套服务能力。推进文化发展“N个一”工程，培育规上文化旅游企业8家，研发文化旅游产品70种以上，积极发展生态养生、森林体验等新兴业态，打造丝绸之路经济带国内外知名文化旅游休闲养生目的地。全市旅游接待人数、综合收入增速高于全省平均水平。

提档升级商贸物流业。支持批零住餐、交通运输等传统服务业改造升级，加快冷链、仓储、配送等现代物流业发展，促进商贸繁荣。完善城乡流通体系，推进平凉南铁路综合性货场建设，提升陇东农副产品批发交易市场、静宁金果博览城等重点市场的集散功能，打造平凉特色小吃产业园，建成农产品批发市场2个、农贸市场3个、冷链物流项目5个。健全县、乡、村电商服务体系，华亭、崇信争创国家级电子商务进农村综合示范县（市），规范提升乡村电商服务站（点），全市电商交易总额增长25%以上。加强平凉养生商贸城、惠民大厦、恒和国际等设施配套和运营管理，促进楼宇经济快速发展。顺应城乡居民需求新变化，发展休闲康养、动漫创意、信息咨询、服务外包等新业态，培育网络消费、体验消费、自驾游等消费新热点，推进消费提档升级。全市第三产业增加值增长7%。

（四）强化扶持优化服务，发展壮大民营经济。坚持“两个毫不动摇”，降低门槛，破除障碍，完善政策，创优环境，着力激发微观主体活力。

放宽市场准入。坚持“非禁即入”，开展市场准入限制专项清理，坚决拆除有碍民营经济发展的“隐形门槛”。持续开展“减证便民”行动，健全全程代理服务机制。推行“证照分离”改革，落实“一照多址”“一址多照”，打破市场主体住所和经营场所限制。实行市场准入负面清单制度，解决“准入不准营”的问题。支持中小企业在产业链、供应链、价值链中找准位置，公平平等参与资源开发、产业培育等领域的混合所有制改革，鼓励民间资本在医疗、养老、教育等民生领域投资兴业。全市新增非公经济市场主体1.5万户。

破解融资难题。强化政银企对接，建立政银企担信息共享平台，“一对一”实施百户民营企业金融帮扶。健全融资担保机制，整合惠民、中小企业信用担保中心资源资产，重组市级担保中心，扩充甘肃金控平凉融资担保公司资本金，提升担保能力。发挥小微企业“助保贷”和农业互助担保基金作用，引导金融机构建立适合民营经济的授信审批、信用评级、客户准入制度，提高土地、林权、知识产权等抵质押率，扩大面对民营企业信贷投放。加强征信体系建设，建立更加高效、便捷的贷款流程。支持民营企业通过发行企业债、公司债、私募债等进行直接融资。

创优营商环境。深入开展“转变作风改善发展环境建设年”活动，认真落实省市支持非公经济发展政策，全面落实各项减税降费措施。深化“双随机一公开”监管，减少对企业的各类检查。清理规范行政审批中介服务，完善涉企收费目录清单制度，加强涉企市场价格行为监管，坚决砍掉不合法不合理的涉企收费事项，禁止垄断性行业指定经营、强制服务、强行收费。建立非公市场主体投诉协调解决机制，积极回应企业合理诉求，坚决防止执行政策“一刀切”行为。着力构建亲清新型政商关系，大力弘扬企业家精神，保护民营企业家合法权益、人身安全和财产安全，让企业家专心创业、放心投资、安心经营。

（五）强化中心城市辐射带动，加快推进新型城镇化进程。坚持系统谋划、多元筹资、民生优先、保障重点，加快发展以人为核心的新型城镇化。全市城镇化率提高1.5个百分点左右。

强化规划引领。启动编制平凉市“多规合一”城乡统筹总体规划，开展中心城区控制性详细规划动态维护，编制中心城区给水、排水（污水）专项规划，分层次开展中心城区重点区域详细城市设计，加快推进县城、乡镇和村庄规划编制，不断完善覆盖城乡的空间规划体系。加强建筑设计管理，挖掘文化历史内涵，提升建筑品质，塑造城市特色风貌。加大城乡规划督察力度，强化规划监管，坚决整治违法建设，切实维护规划的严肃性和权威性。

提升城市承载能力。老城区坚持问题导向，新城区坚持目标导向，投资100亿元，实施3000万元以上中心城区建设项目5大类28项。突出居住环境改善，积极争取棚改专项债券配额，着力破解土地房屋征收难题，全市实施棚户区改造2.5万户，中心城区实现市建司家属院、汪家园西片等10个片区棚改清零，启动西三里塬、兴北路西、天门塬3个片区棚户区改造，完成东西大街、崆峒大道等13个片区1.4万户老旧住宅楼棚改工程；突出基础设施配套，力争开建天门塬规划区“两纵三横”5条干道，建成新民北路、工业园区泾河大道等9条道路，建成天然气支线管道工程，配套建设一批公共停车场，加快城市公园绿地建设和街巷道路、单位小区、生态停车场绿化亮化；突出精细化管理，加快建设城市管理基础数据库，推进智慧城市建设，强化城市综合执法，规范物业服务行为，下功夫提升市政设施、市容市貌、城市交通等管理水平。加快推进县城扩容提质，抓好四十里铺、安口、什字、南湖等特色小镇建设，发挥示范引领作用。

加强城市资源资产经营运筹。建立中心城区可经营性城市资源资产清单，优化中心城区国有资产经营公司，依法授权经营停车场、户外广告、国有资产等资源资产。强化规划财富功能，完善“土地出让+城市设计”制度，增强开发区域增值预期。把依法监控土地作为经营城市的突破口和重点，全面清理“五未”土地，加快棚改腾退土地出让，盘活存量土地资源。

加快农业转移人口市民化。优化户籍迁移政策，实现具备基本落户条件的外来人口落户无门槛、迁入无障碍。推进城镇基本公共服务常住人口全覆盖，保障进城人员随迁子女接受义务教育、学前教育，完善外来务工人员社会保险关系转移接续。坚持“房子是用来住的，不是用来炒的”定位，健全住房市场体系和住房保障体系，统筹解决进城农民工、城镇中低收入居民的住房问题。大力发展城镇产业，支持建成一批工业带动、资源开发、商贸流通、旅游度假型示范城镇，推进产城融合发展，让进城人员进得来、留得住、能发展。

（六）打好污染防治攻坚战，持续加强生态文明建设。扛牢生态文明建设政治责任，提升全民环保意识，建立源头严控、过程严管、后果严惩制度，巩固国家生态文明建设示范市创建成果。

综合实施蓝天碧水净土工程。实施平凉电厂、崇信电厂2台机组超低排放改造，巩固提升燃煤锅炉综合整治成效，精细化管控建筑工地、煤炭市场，切实提高空气环境质量；严格落实河（湖）长制，开建泾河、葫芦河流域水环境综合整治3个项目，完成华亭、灵台、崇信、庄浪城区生活污水处理厂提标扩容改造，加快工业集中区污水处理设施建设，建成36个重点乡镇污水垃圾处理设施，配套完善乡镇污水收集管网，破解运营难题，确保达标处理；落实畜禽养殖禁养区禁养措施，实施土壤污染治理与修复试点项目，实现化肥农药使用量零增长。深化全域无垃圾专项治理，继续开展十大环境卫生治理行动，积极推进餐厨废弃物资源化集中处理，崆峒区、华亭市、崇信县达到省上全域无垃圾治理验收标准。

全面加强生态建设与保护。继续做好县域生态环保红线划定工作，全面实施大规模国土绿化行动，加快国家森林城市创建，加强重点流域生态修复保护，推进三北五期、天保二期、新一轮退耕还林等重点林业生态工程建设，持续抓好城市、园区、景区、道路、河流、庭院等重点区域绿化，城乡造林50万亩，治理水土流失230平方公里，实现生态资源保育保值和生态资本增值积累。

着力强化生态环境执法监管。建立健全环保督察机制，深入实施“绿盾”自然保护区监督检查专项行动，持续抓好突出环境问题整改。加快建设天地空一体化生态环境监测网络管理平台，提升生态环境监测能力。严肃查处偷排偷放、私设暗管、监测数据弄虚作假、不正常使用环保设施等问题，始终保持对环境违法行为的高压态势。

（七）大力推进改革开放创新，激发发展活力动力。坚持问题导向，扭住关键环节，全面落实中央和省市委各项改革举措，以改革增活力，以开放促开发，以创新促发展。

深化重点领域改革。扎实开展“深化放管服改革工作突破年”活动，再取消下放一批行政审批事项，完善各级政务服务中心运行机制，全面提速行政审批效率，工程建设项目审批时限压减至120个工作日以内，市县级政务服务事项网上可办率达到90%以上。持续深化工业园区改革，支持平凉工业园区创建国家高新技术产业开发区。加快推进国企国资改革，全面推行资源资产化、资产资本化、资本证券化改革，健全完善国有企业公司法人治理结构和经营管理机制。深化金融体制改革，围绕建设陇东区域金融中心，加大引行入平力度，完善金融服务体系，大力发展绿色金融。健全政府预算管理，推进财政事权和支出责任划分，加强政府债务管理，规范举债行为，稳妥化解存量债务，严厉打击金融违规违法行为，牢牢守住不发生区域性金融风险和恶性金融事件的底线。全面完成政府机构改革任务。

推进开放交流合作。积极融入“一带一路”和关中平原城市群，充分发挥平凉海关作用，主动对接中国（陕西）自由贸易试验区，全国出口食品农产品质量安全示范区创建通过国家验收。支持外贸龙头企业发展跨境电子商务，在境外建立地方特色产品展销中心、分拨中心，进出口总额增长10%。放大《魅力中国城》竞演成果，抓好典型培育、产品推介、高端论坛等环节工作，精心办好《魅力中国城》走进平凉—第十四届中国牛业发展大会暨崆峒文化旅游节，把“平凉红牛”、平凉旅游等地方特色品牌推向全国。提升金果博览会、西王母华夏母亲节、皇甫谧文化节等节会水平，谋划举办在省内外有影响力的文化旅游活动和体育赛事，以文化大舞台繁荣经贸大市场，着力扩大平凉开放度。

大力实施创新驱动战略。突出企业创新主体地位，支持华煤、红峰、虹光等企业与科研院所建立产学研用长效合作机制，支持天纤棉业、启航未来、荣康实业等创建省级企业技术中心、技术创新企业，平凉海螺水泥创建绿色工厂通过工信部验收。落实“双创”政策，支持科技人员、高校毕业生、退役军人、返乡人士、乡土能人创业就业，支持中小微企业创新创造，市级科技企业孵化器在孵企业达到32户以上。实施质量强市战略，争创一批省级名牌产品和中国驰名商标，有效注册商标4000件以上，专利申请量增长10%。实施市长人才奖，鼓励建立院士（专家）工作站（室），打造高科技人才、企业家、实用人才三支队伍。

（八）着眼普惠共享，切实提升保障和改善民生水平。坚持财力向民生倾斜、政策向民生聚焦、服务向民生覆盖，不断提升广大群众的获得感、幸福感和安全感。

完善就业和社会保障体系。把稳就业摆在优先位置，全面落实新一轮就业创业扶持政策，应届普通高校毕业生就业率达到85%以上。深入推进全民参保计划，积极稳妥推进养老保险制度改革，落实社会保险待遇提标政策。重视加强农民工工作，开展“治欠保支”三年行动计划，创建无欠薪城市。探索医养结合发展模式，完善居家、社区养老服务，提升养老服务质量。有效发挥社会救助兜底作用，做好城乡低收入家庭、特困供养对象、孤儿、高龄和失能老人、残疾人、军烈属生活保障，维护退役军人权益，推进城镇困难职工解困脱困，关爱农村留守儿童、妇女和老人，支持慈善事业发展。

协调发展社会事业。继续调整优化教育布局，扶持办好农村小规模学校和乡镇寄宿制学校，持续增加城镇教育资源，新建改扩建幼儿园20所，全力解决城镇“入园难”“大班额”“择校热”问题。加快平凉职业技术学院三期建设，打造陕甘宁三省区职业教育高地。深化医药卫生体制改革，巩固完善分级诊疗制度，强化基本公共卫生服务，完善以医联体为主要架构的县乡村一体化服务体系，加快16个市县医疗卫生项目建设。实施文化惠民、文艺精品创作、文化遗产保护工程，建成开放市博物馆新馆、市美术馆，新建乡镇（社区）体育中心10个、村级农民体育健身工程200个，丰富群众性文体活动。

创新加强社会治理。深入推进“七五”普法，坚持和发展新时代“枫桥经验”，深化平安平凉建设，严格落实社会治安综合治理领导责任制，完善矛盾纠纷多元化解机制，畅通信访渠道，扎实推进“雪亮工程”，持续开展扫黑除恶专项斗争，依法打击各类违法犯罪活动，构建立体化社会治安防控体系。实施自然灾害防治能力提升工程，加强生产安全、交通安全、环境安全、食品药品安全隐患排查和专项整治，提高应急管理能力和公共安全水平。全面落实党的民族宗教政策，推进民族团结进步示范创建工作，依法加强宗教事务管理，保持民族团结、宗教和顺。

全面加强政府自身建设

各位代表，新形势新任务对政府工作提出了新的更高要求，我们将始终把人民对美好生活的向往作为奋斗目标，按照市委“担当、创新、突破、提升”的工作要求，坚持实字当头、干字为先，以刀刃向内的革新勇气加强自身建设，以只争朝夕的干劲状态推动工作落实。

提高政治站位，自觉自信干。树牢“四个意识”，坚定“四个自信”，坚决做到“两个维护”。坚信只有想不到、没有做不到，不为问题所困、不为困难吓倒，不折不扣推动中央大政方针及省市委各项决策部署落地生根。

规范行政行为，依法依规干。充分发挥法律顾问和专家决策咨询作用，提升政府民主科学依法决策水平。加强和改进政府立法工作，严格规范公正文明执法。依法接受人大法律监督，自觉接受政协民主监督，主动接受社会监督和舆论监督，认真听取各民主党派、工商联、无党派人士和社会各界意见，高质量办好建议提案。深入推进政务公开，切实保障人民群众知情权、参与权、监督权。

勇于担当作为，敢死拼命干。树立底线思维，永葆斗争精神，进一步解放思想，坚决破除精神不振、担当不足的懈怠思想，破除资源禀赋依赖、传统路径束缚的思维定势，破除条块分割、各自为政的狭隘意识，破除庸懒散、推拖绕、等靠要的作风顽疾，以不甘落后的劲头、没有不能的胆识、不怕吃亏的心态、高人一筹的本领，大力构筑精神高地，奋力冲出发展洼地。树立重实干、重实绩导向，健全正向激励和容错纠错机制，完善重点工作清单制度和责任分解机制，加大政务督查督办力度，一件事一件事扭住不放，一锤子一锤子钉在实处，以实实在在的行动推动发展。

坚持廉洁施政，清清白白干。认真落实全面从严治党主体责任，严格落实“一岗双责”，扎实推进政府系统党风廉政建设和反腐败工作，坚决整改巡视、巡查和工作督查反馈问题。从严落实中央八项规定及实施细则精神，坚决整治侵害群众利益的不正之风和腐败问题，以建设清廉政府的实际成效取信于民。

各位代表，时代赋予重任，奋斗铸就辉煌。让我们更加紧密地团结在以习近平同志为核心的党中央周围，在省委省政府和市委的坚强领导下，振奋精神，锐意进取，埋头苦干，攻坚克难，加快建设绿色开放幸福美好新平凉，以优异成绩庆祝中华人民共和国成立70周年！
